# Low–Normal Thyroid Function Is Not Associated with Either Non-Alcoholic Fatty Liver Disease or with Metabolic Dysfunction-Associated Fatty Liver Disease

**DOI:** 10.3390/life13041048

**Published:** 2023-04-19

**Authors:** Julia Zuarth-Vázquez, Lidia Moreno-Castañeda, Juan Pablo Soriano-Márquez, Alain Velázquez-Alemán, Martha Helena Ramos-Ostos, Misael Uribe, Iván López-Méndez, Eva Juárez-Hernández

**Affiliations:** 1 Internal Medicine Department, Medica Sur Clinic & Foundation, Mexico City 14050, Mexico; juliazuarth@gmail.com (J.Z.-V.); lidiamorenoc@gmail.com (L.M.-C.); 2 Gastroenterology and Obesity Unit, Medica Sur Clinic & Foundation, Mexico City 14050, Mexico; jpsoriano92@gmail.com (J.P.S.-M.); muribe@medicasur.org.mx (M.U.); 3 Translational Research Unit, Medica Sur Clinic & Foundation, Mexico City 14050, Mexico; alainvelazquezaleman@gmail.com; 4 Integral Diagnosis and Treatment Center, Medica Sur Clinic & Foundation, Mexico City 14050, Mexico; mtramos@medicasur.org.mx; 5 Hepatology and Transplants Unit, Medica Sur Clinic & Foundation, Mexico City 14050, Mexico

**Keywords:** thyroid function, steatosis, metabolic syndrome, controlled attenuation parameter

## Abstract

Background: The association of low–normal thyroid function (LNTF) with non-alcoholic fatty liver disease (NAFLD) or metabolic dysfunction-associated fatty liver disease (MAFLD) is controversial; thus, the aim of this study is to determine this association. Methods: NAFLD was evaluated by controlled attenuation parameter of transient elastography. Patients were classified by MAFLD criteria. LNTF was defined as TSH levels of 2.5 to 4.5 mIU/L and were divided into three different cut-off points (>4.5 to 5.0, >3.1, and >2.5 mIU/L). Associations between LNTF, NAFLD, and MAFLD were evaluated by univariate and multivariate logistic regression analyses. Results: A total of 3697 patients were included; 59% (*n* = 2179) were male, and median age and body mass index were 48 (43–55) years and 25.9 (23.6–28.5) kg/m^2^, respectively, and 44% (*n* = 1632) were diagnosed with NAFLD. THS levels of 2.5 and 3.1 showed significant associations with the presence of NAFLD and MAFLD; however, LNTF did not show an independent association with the presence of NAFLD or MAFLD in multivariate analysis. According to different cut-off points, patients with LNTF presented similar risks for NAFLD as the general population. Conclusion: LNTF is not associated with NAFLD or MAFLD. Patients with high LNTF are equally at risk for NAFLD as the general population.

## 1. Introduction

Non-alcoholic fatty liver disease (NAFLD) has become the most common liver disorder, currently affecting about 25% of adults worldwide, with a growing prevalence. Although NAFLD is more frequent in obese patients and people with type 2 diabetes mellitus (DM), in addition to being considered the hepatic component of metabolic syndrome (MS), some non-obese patients can also develop NAFLD with a known prevalence of 5% to 30%; the heterogeneity of this prevalence could be explained by a cut-off of body mass index (BMI) to determine overweight [1,2]. Recently, an expert panel proposed a new definition for this disease, re-named as metabolic dysfunction-associated fatty liver disease (MAFLD) with a specific diagnostic criteria for overweight and lean patients, focused in metabolic abnormalities related to liver steatosis [3].

Endocrinopathies such as hypothyroidism, hypopituitarism, hypogonadism, and polycystic ovary syndrome, of which there is less knowledge about the pathophysiological and molecular mechanisms involved in steatogenesis, are emerging risk factors for NAFLD [4,5]. In 1900, von Noorden recognized the relationship between abnormal thyroid function and the development of NAFLD [6], and it was not until 1930 that the relationship between hypercholesterolemia and thyroid dysfunction was concluded. Thyroid hormones, triiodothyronine (T3), and thyroxine (T4) play an important role in the regulation of energy and lipid homeostasis [7]. Most of the key genes involved in these metabolic pathways are regulated by the thyroid hormone receptor-β, the most abundant isoform in hepatocytes that mediates the effects of thyroid hormones on hepatic carbohydrate and lipid metabolism, and novel selective thyroid hormone receptor-β agonists have recently shown beneficial effects on metabolic alterations, including NAFLD [8,9]. 

Specifically, thyroid hormone receptor-β is highly expressed in the liver regulating the metabolism of cholesterol and carbohydrates; the thyroid hormone/thyroid receptors axis is a strong inducer of hepatic autophagy contributing to lipid droplet degradation causing the removal of damaged mitochondria and reactive oxygen species, preventing hepatic injury; and the dysfunction of this axis is related to several metabolic pathologies. In MAFLD, the deiodinase family members 1 and 2 increases T3/T4 levels as hepatic enzymes. Conversely, deiodinase members 3 inactivates TH. Increased Dio1 level promotes β-oxidation of fatty acid and oxidative phosphorylation, then preventing hepatocyte steatosis. It has been observed that the mutation of the TRβ gene in thyroid hormone resistance patients leads to the impairment of TRβ signals in the hepatic steatosis. The mutation type of the TRβ gene is the substitution of glycine by arginine at position 243 (R243Q) of TRβ. Gene mutations may be used to discover the region that regulates TR lipid metabolism in the hinge region, as well as other regions and their functions, and this pathway could be part of NAFLD development [10].

Impairment in the function of the thyroid gland comprises a spectrum of disorders, ranging from asymptomatic to symptomatic thyroid disease. Overt hypothyroidism is characterized by elevated serum thyroid-stimulating hormone (TSH) and decreased free thyroxine levels (FT4), while subclinical hypothyroidism is characterized by elevated serum TSH levels and normal FT4 levels. The terms “subclinical” or “overt” are used in reference to laboratory parameters, because the symptoms can occur when subclinical hypothyroidism is present; on the other hand, symptoms of overt TD are often non-specific and may be unrecognized and unreported by patients. The generally accepted reference range for normal serum TSH is 0.40–4.2 mIU/L, although the normal reference range for TSH varies depending on the laboratory and/or the reference population surveyed, and the range may widen with increasing age and BMI [11,12]. Most sources report higher limits of normal TSH in adults, ranging from 4.0 to 5.5 milliunits per liter (mU/L) and a higher limit of 6.0 mU/L in older adults (>65 years of age). Based on recent meta-analyses [13,14], a “strict-normal” thyroid function is defined as TSH levels between 0.4–2.5 mIU/L and normal FT4 (0.9–1.14 ng/dL); on the other hand, “low–normal” thyroid function (LNTF) is defined as TSH levels between 2.5 mIU/L and 4.5 mIU/L with normal FT4. 

Overt hypothyroidism affects about 3% of the general population, whereas subclinical hypothyroidism affects 5–10% of people, and 8–18% of people older than 65 years [15]. The prevalence of hypothyroidism in patients with NAFLD has been reported from 15% to 36%. Importantly, subclinical hypothyroidism and LNTF have been reported to be associated with MS and cardiovascular mortality, and may not only accelerate hepatocellular lipid accumulation, but could also negatively impact the prognosis of NAFLD, increasing the risk of hepatic and extrahepatic complications [16].

Several studies have shown that thyroid diseases, mainly primary hypothyroidism, are associated with NAFLD; recently, the term “hypothyroidism-induced NAFLD” has been suggested [17]. The Rotterdam study, a large population-based study of 9419 subjects in the Netherlands, showed that elevated TSH levels were significantly associated with NAFLD risk after adjusting for age, sex, alcohol consumption, smoking, and follow-up time for 10 years (OR = 1.09; 95% CI: 1.01–1.19, *p* < 0.05); however, this association became statistically insignificant after adjusting for total cholesterol, triglycerides, BMI, arterial hypertension, DM, and use of lipid-lowering medications (OR = 1.07; 95% CI: 0.98–1.17; *p* > 0.05) [7,18]. 

These results open a discussion about whether adjusting the TSH level to a lower reference could prevent the emergence of NAFLD. The correlation between thyroid hormones within the euthyroid range and NAFLD has not yet been clarified. The heterogeneity in defining thyroid dysfunction, the demographic characteristics of the populations studied, and the conventional abdominal ultrasound used as the diagnostic method for NAFLD in most studies bring controversial results. Nowadays, there are no studies evaluating the association between LNTF and NAFLD using transient elastography (TE) with controlled attenuation parameter (CAP) as a diagnostic method for NAFLD, and there is less evidence about the association of LNFT with MAFLD. The aim of this study is to establish if there is an association between LNTF with NAFLD and MAFLD, as determined by CAP. 

## 2. Materials and Methods

### 2.1. Patient Population 

An observational, comparative, cross-sectional, retrospective study was carried out. All the clinical files of the adult patients who attended a preventive check-up from January 2019 to October 2020 at the Integral Center for Diagnosis and Treatment of the Medica Sur Hospital were reviewed. The records of adult patients who had complete clinical, anthropometric, biochemical, and TE assessment data were included. Demographic variables, hereditary family history, and pathological personal history of chronic-degenerative diseases were collected. MS was defined according to Adult Treatment Panel III. Laboratory studies included blood count, blood chemistry, lipid profile, and liver function tests taken from blood samples after fasting for at least 8–12 h. Fasting glucose, lipid profile, and liver function tests were measured by enzymatic kinetic method (Beckman Coulter DXC800, Brea, CA, USA), HbA1c was determined by ion exchange HPLC (BioRad D10, Hercules, CA, USA), and ultrasensitive c-reactive protein was determined by immunoturbidimetry (Beckman CoulTer DXC800). 

Records of patients with a previous diagnosis of liver disease other than NAFLD, such as viral hepatitis (hepatitis B or C virus), autoimmune hepatitis, hereditary (Wilson, hemochromatosis, alpha 1-anti-trypsin deficiency) and decompensated liver cirrhosis were excluded; patients with significant alcohol consumption (men, more than 21 drinks per week = more than 210 g per week or more than 30 g per day, and women, more than 14 drinks per week = more than 140 g per week or more than 20 g per day) or those with the human immunodeficiency virus infection were also excluded. 

### 2.2. Low Normal Thyroid Function Definition and Biochemical Evaluation

For this study, TSH levels were measured using the third-generation immunochemiluminescence method (Beckman Coulter DXI800), with functional analytical sensitivity, with a minimum cutoff < 0.003 mIU/L and a maximum cutoff > 500 mIU/L. LNTF was defined as TSH levels between 2.5 and 4.5 mIU/L, based on meta-analysis thyroid function tests ranges [12,13]. Then, we established three cut-off points of LNTF: >2.5 mIU/L; >3.1 mIU/L (percentile 75); and 4.5 to 5.0 mIU/L to evaluate the association of these levels with the presence of liver steatosis.

### 2.3. Liver Disease Assessment

The presence of liver steatosis was determined by TE (FibroScan^®^, Echosens^TM^, Paris, France), with CAP. With fasting for at least 4 h, it was performed by a single expert operator in the TE technique, using the M or XL probe according to BMI > 27 kg/m^2^. Ten measurements were considered valid, with an IQR-CAP <40 and <30 for liver stiffness; studies not meeting the reliability criteria were excluded. Patients with significant liver fibrosis (>8.0 kPa) were excluded for the analysis. The determination of steatosis was established according to Sirli et al. cut-off points as follows: S0, <263 dB/m; S1, 263–282 dB/m; S2, 283–295 dB/m; and S3, >296 dB/m [19].

NAFLD was defined by the presence of steatosis according to CAP; meanwhile, MAFLD was determined according to Eslam et al. definition as evidence of liver disease detected by CAP and overweight/obesity or DM; in lean patients, MAFLD was determined as evidence of liver steatosis detected by CAP and the presence of at least two metabolic risk abnormalities [3].

### 2.4. Anthropometric Measurements

Anthropometric, waist, and hip circumference data in centimeters were obtained by a standardized nutritionist. Body fat percentage and weight in kilograms were measured by electrical impedance (Tanita Corporation, Tokyo, Japan). 

### 2.5. Statistical Analysis

Data distribution was evaluated using the Kolmogorov–Smirnov test, resulting in non-parametric, for which continuous variables are expressed as medians and interquartile ranges. Categorical variables are described with percentages and numbers. For association between TSH levels and NAFLD/MAFLD (adjusted by BMI), an univariate analysis was performed and those variables with a *p* value ≤ 0.10 were included in the multivariate analysis by logistic regression to determine the independence of the associations. A *p* value ≤ 0.05 was considered statistically significant. All statistical analyses were performed using the statistics program SPSS/Mac v.25 (SPSS, Chicago, IL, USA).

## 3. Results

A total of 3697 patients were included. Among them, 59% (*n* = 2179) were male, the median age was 48 [43–55] years, and BMI was 25.9 [23.6–28.5] kg/m^2^. A total of 60% (*n* = 2238) of patients presented as overweight; according to ATP III criteria, 19.7% (*n* = 727) had MS. DM was identified in 6.2% (*n* = 230) of patients. LNTF was found in 37.6% (*n* = 1389), THS levels >4.5 were observed in 7.7% (286) of patients. According to CAP, liver steatosis was present in 44% (*n* = 1632) of patients, showing a higher prevalence of S3 (54.2%, *n* = 871), whereas 1.6% (*n* = 59) presented liver fibrosis (F1-F2, 6.0 to 7.9 kPa). MAFLD was identified in 1432 overweight patients (38.7%) and in 13.8% (*n* = 510) lean patients of a total of 1460. General characteristics of patients are presented in Table 1. 

Of the 1389 patients with LNTF, 46.7% (*n* = 648) were diagnosed with NAFLD; 31% (*n* = 202), S1; 15% (*n* = 96), S2; and 54% (*n* = 350), S3. It is important to mention that 62% (*n* = 861) were overweight (BMI > 25 kg/m^2^). 

FT4 levels were only available in 476 patients to determine the prevalence of subclinical and overt hypothyroidism. Of these patients, 7.1% (*n* = 34/476) had TSH >4.5 mIU/L, and 3.4% (*n* = 16/476) were classified as subclinical hypothyroidism with normal FT4, and 3.8% (*n* = 18) with overt hypothyroidism (FT4 < 0.9 ng/dL). The prevalence of NAFLD in patients with subclinical hypothyroidism was 43.8% (*n* = 7), most of them S3 (85.7%, *n* = 6); on the other hand, NAFLD was present in 38.9% (*n* = 7) of patients with clinical hypothyroidism, 71.4% (*n* = 5) of whom were S3. The presence of overt hypothyroidism did not show a significant association with NAFLD (OR 1.26 (CI 95% 0.80–2.28, *p* = 0.47) or MAFLD (OR 1.10 (CI 95% 0.61–1.99), *p* = 0.81), and neither did those with subclinical hypothyroidism (OR 1.26 (CI 95% 0.63–1.97), *p* = 0.80 and OR 1.4 (CI 95% 0.61–1.99), *p* = 0.79).

Regarding the association of LNTF with NAFLD (*n* = 3697), TSH >2.5 and >3.1 were associated in univariate analysis (OR 1.0 (CI 95% 1.0–1.1), *p* = 0.01 and OR 1.1 (95% CI 1.0–1.2), *p* = 0.004, respectively). THS levels >4.5 did not show significant associations; however, none of the three TSH cut-off points showed an independent association in multivariate analysis (Table 2).

Once again, when patients were evaluated by MAFLD criteria (overweight/obese or DM), TSH > 4.5 did not show significant associations; meanwhile, TSH > 2.5 (OR 1.0 (CI95% 1.0–1.1), *p* = 0.04) and TSH >3.1 (OR 1.0 (CI95% 1.0–1.1), *p* = 0.02) showed a significant association in univariate analysis, but not so in multivariate analysis (Table 3).

In lean MAFLD patients, AST > 26.9, ALT > 30.4, fat percentage > 29.8%, waist circumference > 92 cm, and waist/hip ratio > 0.92 were associated with MAFLD, and only ALT (OR 1.9 (CI95% 1.0–3.6), *p* = 0.03) levels and fat percentage (OR 3.4 (CI95% 1.6–7.0), *p* = 0.001) showed independent associations; the three cut-off points of TSH levels did not show significant associations. 

We carried out univariate and multivariate sub analyses in order to determine NAFLD-associated factors in the three different cut-off points of TSH levels and we observed that these were similar to the general population, meaning, the presence of MS, ALT > 30.4, and fat percentage > 29.8% of those with independent associations in all TSH cut-off points (Table 4).

## 4. Discussion

Even though there are several reports about associations of TSH, FT3, and FT4 with NAFLD, there is a high heterogeneity in results that could be explained by different TSH levels cut-off points to define LNTF, as well as NAFLD diagnostic methods. This study has established three different cut-off points for TSH for LNTF in euthyroid patients to evaluate the association with two definitions for fatty liver disease, both diagnosed by CAP, that have shown a better sensitivity (66–80% according to grades of steatosis) than other screening methods, such as abdominal ultrasound (60%) or biochemical indexes (30–70%) [20,21,22], and the diagnostic accuracy is not lower compared with the biopsy (gold standard), especially in advanced stages of liver steatosis [23,24]. On the other hand, serum TSH levels were measured by third generation immunochemiluminiescence, a non-isotopic method, that uses constant amounts of two antibodies. The first—in the labelled reactant, monoclonal mice anti-TSH antibodies labelled with acridine ester—and the second—in the solid phase, labelled with acridine ester, polyclonal sheep anti-TSH antibodies covalently linked to paramagnetic particles. This method achieves functional sensitivity (FS = ≤0.01 mIU/L) [25,26]. With this methodology, our results did not show independent associations between LNTF and the presence of NAFLD or MAFLD.

The prevalence of increased levels of TSH has been analyzed in different studies. In a retrospective cohort of 18,544 subjects who attended a preventive check-up in Korea, Lee et al. did not observe a statistically significant difference in TSH levels between the groups with and without NAFLD.18 In results of cross-sectional studies performed in Iran [27], Turkey [28] and Germany [29], the biggest one with 1276 patients, significant differences of TSH levels have not been observed regarding the presence of NAFLD. Moreover, results were different in two meta-analysis; the first one in 2018, with 26 studies and 61,548 patients, observed significantly higher TSH levels in patients with NAFLD and an increase in TSH in patients with progression of NAFLD. These results were disputed in subsequent evaluations based on the degree of hypothyroidism and might be inconsistent due to the small number of studies included; instead of thyroid hormones, THS might play a key role in the onset and progression of NAFLD.17 In Zeng et al. meta-analysis, performed in 2021, also higher TSH levels have been observed in NAFLD patients; however, the conventional abdominal ultrasound was used as the diagnostic method for NAFLD in 9 of the 10 studies and liver biopsy in one of them [13]. In our study, patients with NAFLD and MAFLD also showed significantly higher levels of TSH (>2.5 and >3.1 cut-off points). High TSH levels have also been observed in DM patients but, once again, the results are inconsistent even in similar populations. Wang et al. conducted a cross-sectional study of 400 hospitalized patients with DM in China. They found that TSH levels were significantly higher (*p* = 0.02) than in patients with DM but without NAFLD [5].

Despite these observations, when high TSH levels were associated with risk for NAFLD, results have also been inconsistent. In their prospective cohort of 9419 subjects from the Rotterdam study in the Netherlands, Bano et al. showed that elevated TSH levels were significantly associated with NAFLD risk after adjusting for age, sex, alcohol consumption, smoking, and follow-up time (OR = 1.09; 95% CI: 1.01–1.19, *p* < 0.05); however, this association became statistically insignificant after adjusting for total cholesterol, triglycerides, BMI, arterial hypertension, DM, and use of lipid-lowering medications (OR = 1.07; 95% CI: 0.98–1.17; *p* ≥ 0.05). With a population of 10,539 subjects in Brazil, Janovsky et al. [30] showed that higher TSH levels were associated with a higher prevalence of NAFLD among euthyroid subjects, after adjusting for age and gender (OR = 1.22, *p* < 0.01). Nonetheless, this association was not significant when adjusting for MS characteristics (waist circumference, triglycerides, HDL-C, blood pressure, fasting glucose) and smoking (OR = 0.93; *p* = 0.20). On the other hand, Tahara et al. [31] evaluated 140 subjects in Japan, observing that TSH elevation was an independent risk factor for NAFLD after adjusting for metabolic risk factors (BMI, HDL-C, triglycerides, arterial hypertension, and DM) in the multivariate analysis (OR = 1.12, 95% CI: 1.01–1.40, *p* = 0.033); however, BMI cut-off points need to be taken into account carefully. In our study, three different cut-off points of TSH levels only showed an association with NAFLD in univariate analysis; independent associations have not been observed.

There is less evidence about the association of TSH levels in MAFLD due to the recent introduction of the criteria. In a recent study of 18,427 euthyroid patients, Fan et al. observed that TSH levels were higher in MAFLD patients (1.98 ± 0.90 vs. 1.95 ± 0.90, *p* = 0.020); higher TSH levels (Q3 and Q4) were associated with MAFLD. When they classified patients according to MAFLD type (diabetes type, overweight/obesity type, and metabolic disorder type), this association was maintained only in overweight/obesity MAFLD type, concluding that the association between thyroid hormones and MAFLD is mediated by being overweight. The presence of liver steatosis was diagnosed by abdominal ultrasound [32]. In our study, high TSH levels did not show an independent association with MAFLD, when this was evaluated by CAP. Nonetheless, the association of high TSH levels as risk factor for MAFLD is not clear yet, particularly when this new definition has a stricter metabolic criteria. A recent study in Asian populations observed that elevated concentrations of TSH are associated with higher risk of liver fibrosis in patients with NAFLD, even in euthyroid state. Despite the large sample size (19,946 patients), it is important to take into account that MAFLD was determined by abdominal ultrasound, whereas liver fibrosis was estimated by biochemical markers [33]. A high risk of liver fibrosis in MAFLD patients with elevated TSH levels was also observed in the study by Zhang et al. in 776 euthyroid patients with DM [34].

The pathophysiological mechanisms that have been studied to explain the association of TD and NAFLD are insulin secretion and lower hepatic β-oxidation, which favors the accumulation of triglycerides and lipid mediators such as diacylglycerols, sphingomyelins, and ceramides that induce insulin resistance, impaired suppression of gluconeogenesis, and stimulation of de novo lipogenesis in the liver [35]. In early studies of murine models, TD was shown to reduce lipolysis in adipose tissue due to a decreased insulin secretion and an increased flux of free fatty acids to the liver [36].

TSH was shown to be involved in the homeostasis of appetite-regulating hormones from adipose tissue, such as leptin, as TSH stimulates its secretion with a direct effect on adipocytes. Hyperphagia with the consequent body weight increase exacerbates triglyceride accumulation in the liver. In addition, higher TSH levels can induce steatosis via TSH receptor (TSHR) signaling, by also increasing hepatic gluconeogenesis, repressing hepatic bile acid synthesis, and causing hypercholesterolemia by decreasing 3-hydroxy- 3-methylglutaryl-CoA reductase phosphorylation. Furthermore, higher levels of proinflammatory adipokines contribute to hepatic inflammation and fibrosis [37,38].

Although the role of TD in the pathogenesis of NAFLD, together with MS components, is biologically plausible, there is still a lack of data supporting this association. First, high–normal TSH levels are associated with a high BMI [39]. Obesity is closely related to NAFLD due to the ectopic fat accumulation in the liver and hepatic insulin resistance, and insulin resistance has also been found in hypothyroidism. It is important to mention that in our study, 62% (*n* = 861) of the 1389 patients with LNFT were overweight (BMI > 25.9 kg/m^2^) and the prevalence of NAFLD was higher than 40%, with advanced stage (S3) in most patients.

However, information on thyroid hormone levels in all these studies was collected at the same time point or at baseline; therefore, the results are limited to drawing strong conclusions on the association between changes in TSH and incidental NAFLD. LNTF can be transient or permanent and the study of TSH in serum is not perfect, since it has the potential to interfere with laboratory analyses; results must be interpreted according to the patient’s clinical context, compared with previous and repeated results. Therefore, because of its retrospective nature, a limitation in our study is that we lacked information about the consumption of drugs or supplements that may interfere with various steps of thyroid hormone metabolism that may increase the risk for hypothyroidism, including a history of previous surgery or radiation therapy on the thyroid gland or head and neck area, the presence of anti-thyroglobulin antibodies, and secondary or tertiary hypo/hyperthyroidism causes.

The United States Preventive Services Task Force (USPSTF) concludes that the current evidence is insufficient to assess the balance of benefits and harms of screening for thyroid dysfunction in nonpregnant, asymptomatic adults [11]. Nevertheless, people with subclinical hypothyroidism should undergo screening for NAFLD.

After correcting the mixed factors such as BMI, the results indicated that the risk of NAFLD with TSH levels above 2.5 was the same as for the general population. Higher (but lower than upper limit) levels of AST and ALT were independently associated with risk of NAFLD in LNTF patients. Statistically significant positive linear trends have been noted between elevated AST, ALT, and alkaline phosphatase concentrations and the presence of increasing numbers of metabolic abnormalities [40]. The exact pathophysiological mechanism of how increased liver markers are related to MS remains elusive. One possible explanation is that elevated liver markers are indicative of excess fat deposition in the liver, which is a characteristic sign of NAFLD. Another possible explanation is that elevated liver enzymes indicate liver inflammation, as suggested by Nakanishi et al. [41]. Still, further, Patel et al. reported that liver enzymes and MS risk may be linked with NAFLD and hepatic insulin resistance, in addition to excess visceral adiposity. The association of liver enzymes abnormalities and thyroid function has been described in patients with hypothyroidism in which higher levels of ALT and AST has been related with diminished lipid metabolism and hypothyroidism-induced myopathy, respectively, both conditions related to NAFLD development; however, in LNTF patients this association has not been fully described [42].

Early detection of abnormal thyroid function is of clinical relevance because people with subclinical hypothyroidism have a higher likelihood of progression to overt hypothyroidism than those with TSH levels <4.5 mIU/L. People with subclinical hypothyroidism should undergo screening for NAFLD as well [43]. The use of transient elastography as a diagnostic method for NAFLD to accurately measure hepatic steatosis and fibrosis, even in mild stages, represents a strength in our study. The discrepancies in the previous studies have been mainly due to the method used to diagnose NAFLD, the heterogeneity in defining LNTF, the demographic characteristics of the populations studied, the sample size, and the observational design of most studies, which complicates the comparison in order to determine an independent association.

There have been many studies on NAFLD and thyroid hormone, but the correlation between thyroid hormones within the euthyroid range and NAFLD has not yet been clarified. At present, fewer studies have explored the association between thyroid hormones and NAFLD severity in euthyroid subjects. In this study, we investigated the relationship between LNTF and the occurrence of NAFLD in euthyroid individuals with the aim of providing insights into new predictors and treatment targets for NAFLD.

As strengths, in our study, liver steatosis was determined by CAP, which have a similar diagnostic accuracy than magnetic resonance and higher than other non-invasive methods, specifically abdominal ultrasound, of which results are operator dependent. On the other hand, for our patients who do not have overt hypothyroidism, an endocrinopathy may make the association with NAFLD clearer, but it is still controversial.

## 5. Conclusions

LNTF was not independently associated with the presence of NAFLD or MAFLD. Patients with LNTF have the same risk for NAFLD as the general population. Further large clinical trials are required to identify more modifiable risk factors for NAFLD.

Low thyroid function is a phenomenon that could not be related with NAFLD; nonetheless, the presence and severity of other metabolic abnormalities (primarily obesity) associated with the development of NAFLD could increase TSH levels, increasing the risk for NAFLD due to interplay mechanisms of thyroid and liver dysfunction. It is unknown if these interplays are linear or bidirectional; however, we observed that a high percentage of body fat is an independent factor associated to NAFLD in increasing cut-off points of TSH. These patients could have an increased risk of progression of liver disease to steatohepatitis and fibrosis; therefore, follow-up studies are needed to elucidate the relationship between LNTF and NAFLD. 

## Figures and Tables

**Table 1:** Clinical, anthropometrical, and biochemical characteristics of patients (*n* = 3697).

Characteristic	% (*n*)/M [IQR]
Male	58.9 (2179)
Age	48 [43–55]
Smoking	20.6 (760)
BMI (kg/m^2^)	25.9 [23.6–28.5]
DM	6.2 (230)
MtS	19.7 (727)
Hypertension	20.7 (767)
BMI > 25 kg/m^2^	60.5 (2238)
Fasting glucose	91 [86–98]
Triglycerides	113 [77–164]
Cholesterol	199 [174–225]
HDL	47 [39–57]
LDL	124 [103–147]
RCP	1.3 [0.60–2.8]
HbA1c	5.4 [5.1–5.6]
ALT	25 [19–35]
AST	24 [21–29]
WC	92 [84–99]
HC	100 [96–105]
W/H Ratio	0.92 [0.85–0.97]
Fat percentage	28.3 [23.5–34]
dB	254 [215–295]
Steatosis	44.1 (1632)
S1	31.9 (521)
S2	14.9 (240)
S3	54.2 (871)
kPa	4.1 [3.5–4.8]
Liver fibrosis (F1–F2)	1.6 (59)
TSH	2.1 [1.4–2.9]
TSH > 4.5	7.7 (286)
TSH > 2.5	37.6 (1389)

BMI, body mass index; DM, diabetes mellitus; MtS, metabolic syndrome; HDL, high density lipoprotein; LDL, low density lipoprotein; RCP, reactive C protein; HbA1c, glycosylated hemoglobin; ALT, alanine aminotransferase; AST, aspartate aminotransferase; WC, waist circumference; HC, hip circumference; W/H, waist/hip; TSH, thyroid stimulating hormone.

**Table 2:** Factors associated with non-alcoholic fatty liver disease (*n* = 3697).

	Univariate		Multivariate	
Factor	OR (CI95%)	*p*	OR (CI95%)	*p*
Male	1.5 (1.4–1.6)	**	1.5 (1.1–2.0)	0.002
MtS	2.0 (1.9–2.1)	**	1.9 (1.5–2.4)	**
TSH > 4.5	0.9 (0.8–1.0)	0.15		
TSH > 2.5	1.0 (1.0–1.1)	0.01		
TSH > 3.1	1.1 (1.0–1.2)	0.004		
BMI > 25 kg/m^2^	3.5 (3.1–3.9)	**	1.7 (1.3–2.1)	**
49 years	1.1 (1.0–1.2)	**		
Cholesterol > 200	1.0 (0.9–1.1)	0.07		
HbA1c > 5.5%	1.4 (1.3–1.5)	**		
AST > 26.9	1.4 (1.3–1.6)	**	1.3 (1.0–1.6)	0.006
ALT > 30.4	1.7 (1.6–1.8)	**	1.4 (1.1–1.8)	**
BMI > 26.5	2.9 (2.7–3.2)	**	2.1 (1.7–2–6)	**
Fat % > 29.8	1.5 (1.4–1.6)	**	2.8 (2.2–3.6)	**
WC > 92	3.0 (2.7–3.3)	**	1.7 (1.3–2.1)	**
W/H ratio > 0.92	2.2 (2.0–2.4)	**	1.8 (1.4–2.3)	**

** *p* ≤ 0.0001; MtS, metabolic syndrome; TSH, thyroid stimulating hormone; BMI, body mass index; HbA1c, glycosylated hemoglobin; AST, aspartate aminotransferase; ALT, alanine aminotransferase; WC, waist circumference; W/H waist/hip.

**Table 3:** Factors associated with metabolic associated-dysfunction fatty liver disease in overweight patients (*n* = 2238).

	Univariate		Multivariate	
Factor	OR (CI95%)	*p*	OR (CI95%)	*p*
Male	1.1 (1.0–1.2)	**	1.5 (1.0–2.1)	0.01
MtS	1.4 (1.3–1.5)	**	1.8 (1.4–2.3)	**
TSH > 4.5	0.9 (0.8–1.0)	0.11		
TSH > 2.5	1.0 (1.0–1.1)	0.04		
TSH > 3.1	1.0 (1.0–1.1)	0.02		
HbA1c > 5.5	1.2 (1.1–1.3)	**	1.2 (1.0–1.4)	0.04
AST > 26.9	1.2 (1.1–1.3)	**	1.3 (1.0–1.7)	0.01
ALT > 30.4	1.3 (1.2–1.4)	**	1.3 (1.0–1.7)	0.02
BMI > 26.5	1.8 (1.6–2.0)	**	2.1 (1.7–2.6)	**
Fat % > 29.8	1.2 (1.1–1.3)	**	2.6 (1.9–3.5)	**
WC > 92	1.8 (1.6–2.0)	**	1.7 (1.3–2.2)	**
W/H ratio > 0.92	1.4 (1.3–1.5)	**	1.5 (1.1–2.0)	0.001

** *p* ≤ 0.0001; MtS, metabolic syndrome; TSH, thyroid stimulating hormone; BMI, body mass index; HbA1c, glycosylated hemoglobin; AST, aspartate aminotransferase; ALT, alanine aminotransferase; WC, waist circumference; W/H waist/hip.

**Table 4:** Factors associated with non-alcoholic fatty liver disease in different cut-off points of TSH adjusted by BMI > 25 kg/m^2^.

	TSH > 4.5 (*n* = 286)				TSH > 2.5 (*n* = 1389)				TSH > 3.1 (*n* = 1007)			
	Univariate		Multivariate		Univariate		Multivariate		Univariate		Multivariate	
Factor	OR (CI95%)	*p*	OR (CI95%)	*p*	OR (CI95%)	*p*	OR (CI95%)	*p*	OR (CI95%)	*p*	OR (CI95%)	*p*
Male					1.1 (1.0–1.2)	0.03			1.1 (0.9–1.2)	0.06		
MtS	1.4 (1.2–1.7)	**	3.0 (1.3–6.8)	0.009	1.4 (1.3–1.5)	**	2.2 (1.5–3.2)	**	1.9 (1.7–2.1)	**	2.2 (1.4–3.4)	**
HbA1c >5.5					1.1 (1.0–1.2)	0.003						
AST >26.9					1.2 (1.0–1.3)	**			1.2 (1.0–1.3)	0.001		
ALT >30.4	1.3 (1.0–1.6)	0.01	2.0 (1.0–4.5)	0.03	1.3 (1.2–1.5)	**	1.9 (1.2–2.9)	0.002	1.3 (1.2–1.5)	**	1.9 (1.2–3.2)	0.006
Fat % >29.8	1.5 (1.1–1.9)	**	5.3 (2.3–12.1)	**	1.2 (1.1–1.3)	**	2.7 (1.7–4.2)	**	1.2 (1.1–1.4)	**	2.7 (1.6–4.5)	**
WC >92	1.4 (1.0–2.0)	0.03			1.7 (1.4–2.0)	**	2.6 (1.7–4.0)	**	1.6 (1.3–1.9)	**	2.3 (1.4–3.9)	0.001
W/H ratio >0.92	1.2 (0.9–1.6)	0.08	2.4 (0.9–6.6)	0.07	1.3 (1.1–1.4)	**			1.2 (1.1–1.4)	**		

** *p* ≤ 0.0001; TSH, thyroid stimulating hormone; MtS, metabolic syndrome; HbA1c, glycosylated hemoglobin; AST, aspartate aminotransferase; ALT, alanine aminotransferase; WC, waist circumference; W/H, waist/hip.

## Data Availability

The data presented in this study are available on request from the corresponding authors. The data are not publicly available due to privacy and ethics.
